# Pangenomics for Agricultural Breeding: Construction Strategies, Evidence Integration, and Translational Constraints

**DOI:** 10.3390/biology15110832

**Published:** 2026-05-25

**Authors:** Jinpeng Shi, Ying Lu, Zhengmei Sheng, Huaijing Liu, Keyu Li, Yuqing Chong, Zhendong Gao, Weidong Deng, Dongwang Wu

**Affiliations:** Yunnan Provincial Key Laboratory of Animal Nutrition and Feed, Faculty of Animal Science and Technology, Yunnan Agricultural University, Kunming 650201, China; sjp020607@163.com (J.S.); yinglu_1998@163.com (Y.L.); 18008847283@163.com (Z.S.); 18361309553@163.com (H.L.); 17787916184@163.com (K.L.); 2022004@ynau.edu.cn (Y.C.); zander_gao@163.com (Z.G.)

**Keywords:** pangenome, graph genome, telomere-to-telomere assembly, structural variants, molecular breeding, trait mapping

## Abstract

Pangenomics has become an important framework for representing genetic diversity beyond a single linear reference genome. In agricultural species, it improves access to structural variants (SVs), copy number variations (CNVs), presence/absence variations (PAVs), and non-reference regulatory or coding sequences that may contribute to domestication, adaptation, and breeding traits. This review summarizes recent progress in long-read sequencing, telomere-to-telomere (T2T) assembly, and graph-based genome analysis, with emphasis on both livestock and crop systems. We first define the conceptual boundary between pangenome representations and reference-based variant catalogs. We then compare three major technical routes: variant integration, reference-guided iterative graph construction, and reference-free graph construction. Their performance is evaluated in terms of accuracy, scalability, coordinate consistency, reference bias, computational demand, annotation transfer, and suitability for downstream breeding questions. We further discuss how pangenome resources support hidden variant discovery, QTL and GWAS interpretation, environmental adaptation analysis, and multi-omics-based candidate prioritization. Importantly, we highlight unresolved limitations, including graph complexity, pipeline-dependent SV calls, incomplete functional annotation, weak cross-study comparability, and the difficulty of distinguishing causal variants from linked or neutral variation. This review therefore treats pangenome studies as connected but non-equivalent evidence: resource-building studies establish representational breadth, method papers define technical feasibility, and trait-focused studies provide varying levels of biological support. Apparent inconsistencies among studies are interpreted as signals of differences in sampling, genome complexity, validation depth, and graph construction strategy rather than as simple disagreements.

## Simple Summary

Traditional genomic analysis often uses one linear reference genome, which can miss non-reference sequences, structural variations, and presence/absence variations that are important for agricultural traits. This review evaluates how pangenome resources are constructed and used in animal and crop breeding research. Rather than treating pangenomics as a single method, we distinguish reference-based variant integration, reference-guided graph construction, and reference-free graph construction, and we compare their practical strengths and limitations. We also discuss how pangenomes can improve trait interpretation when combined with QTL, genome-wide association studies (GWASs), and transcriptomic, epigenomic, and functional evidence. The review emphasizes that most current studies are strongest for variant discovery and candidate prioritization, whereas routine breeding deployment still requires stronger benchmarking, reproducibility standards, and experimental validation.

## 1. Introduction

Animal and plant breeding technologies have evolved from traditional phenotypic selection and molecular marker-assisted breeding to genome-wide selection. While genomic selection (GS) significantly enhances breeding value predictions through genome-wide single-nucleotide polymorphisms (SNPs), a single linear reference genome inherently constrains its overall effectiveness [1]. This limitation severely hampers the in-depth analysis of the genetic mechanisms underlying complex traits. Consequently, this linear model fails to capture the full scope of population-level genetic diversity. Since reference genomes represent the sequence characteristics of only a tiny fraction of individuals, widespread non-reference sequences within species and missing variants often remain unaccounted for in analyses. This reference bias obscures complex genetic variants and compromises the reliability of genomic selection models used to predict superior traits. The concept of the pangenome offers a novel approach to overcoming this bottleneck. By integrating genomic sequences from multiple individuals, the pangenome constructs a comprehensive resource encompassing all genes within a population, providing a new platform for studying genetic variation, population diversity, and trait associations. Thus, the emergence of pangenomics offers novel technical pathways for deciphering the genetic basis of complex traits in animals and plants and advancing precision breeding. To overcome these limitations, the pangenome approach has demonstrated significant advantages in domestication studies [2], trait gene mapping [3], and molecular breeding [4]. Systematically reviewing the application progress of pangenomics in animal and plant genetic improvements is crucial for guiding future molecular design breeding.

The concept of pangenomics was first proposed by Tettelin [5] in their 2005 study of *Streptococcus*. They discovered that a single genome cannot represent the entire genetic content of a species. However, eukaryotic genomes, due to the presence of complex structures such as introns and intergenic regions, are significantly larger and more complex than prokaryotic genomes. Combined with sequencing cost and technical limitations, this led to a later start in eukaryotic pangenome research [6]. Despite these initial hurdles, the rapid advancement of high-throughput sequencing technologies eventually provided the tools needed to overcome these barriers. Since then, pangenome research has expanded from prokaryotes to complex eukaryotic systems. In agricultural species, this shift is especially important because economically important traits are often influenced not only by SNPs, but also by SVs, CNVs, and PAVs [7]. Many studies use 50 bp as an operational threshold for defining SVs, although this boundary is methodological rather than absolute and may vary across platforms and databases [8]. The concept of the plant pangenome was introduced by Morgante et al. [9] in 2007, who proposed that a single plant reference genome cannot capture the full genomic complement of a species because transposable elements and structural variation generate extensive inter-individual sequence diversity. To better capture such complex variations, in 2010, Li et al. [10] utilized second-generation sequencing (NGS) technology to construct the first human pangenome, supplementing the reference sequence and identifying gene sequences unique to Asian populations for the first time. Since then, pangenome research in both animals and plants has gradually progressed. In 2014, pioneering research on wild soybean (*Glycine soja*) first demonstrated that the PAV are associated with important agronomic traits, including disease resistance, flowering time, and seed composition [11]. Subsequently, pangenome studies have been reported in various plant species, such as wheat [12], chili pepper [13], rice [14], sesame [15], and *Arabidopsis* [16]. Moving beyond simple variation cataloging, these pangenomic resources have shifted the focus from a linear-centric perspective to the comprehensive characterization of ‘core’ and ‘variable’ genomic landscapes. This transition has enabled the systematic recovery of structural variants and non-reference sequences previously obscured by single-reference templates, thereby unlocking the vast potential of pangenomes for functional genomics discoveries. Additionally, researchers are dedicated to filling previously unresolved sequence gaps, generating representative T2T genome assemblies to decode the final dark matter long unexplored in complex plant genomes [17]. Although early crop pangenomes such as soybean, rice, wheat, and pepper established that PAVs and non-reference genes are widespread, these studies differed greatly in sample composition, assembly depth, and validation strategy. Thus, their common conclusion is not that every missing sequence is breeding-relevant, but that single-reference analyses systematically under-sample the search space for causal or linked.

In recent years, breakthroughs in Third-Generation Sequencing (TGS) technologies—represented by PacBio and Oxford Nanopore—combined with T2T assembly strategies have collectively propelled pangenome research into a new era of high-quality, comprehensive coverage [18]. Long-read sequencing better bridges complex repetitive regions, while T2T assembly aims to achieve gap-free, complete chromosome assembly. This enables researchers to deeply explore the structure and function of previously challenging regions like telomeres and centromeres [19,20]. This technological breakthrough has yielded landmark achievements across multiple species: for instance, Miga [21] pioneered the T2T assembly of the human X chromosome, mapping methylation patterns across complex tandem repeats and satellite arrays. In livestock research, Cao [22] and Zong [23] achieved T2T-level genome assemblies for Jinhua, Min, and Rongchang pigs, respectively, providing the most comprehensive genomic resources to date for pig pangenome analysis and precision genetic improvement. These advances are directly relevant to pangenome construction because high-quality gap-free assemblies eliminate alignment artifacts associated with fragmented references, providing a highly contiguous foundation that improves the precise anchoring and representation of large insertions, inversions, and other complex structural variants within the pangenome graph.

**Figure 1:**
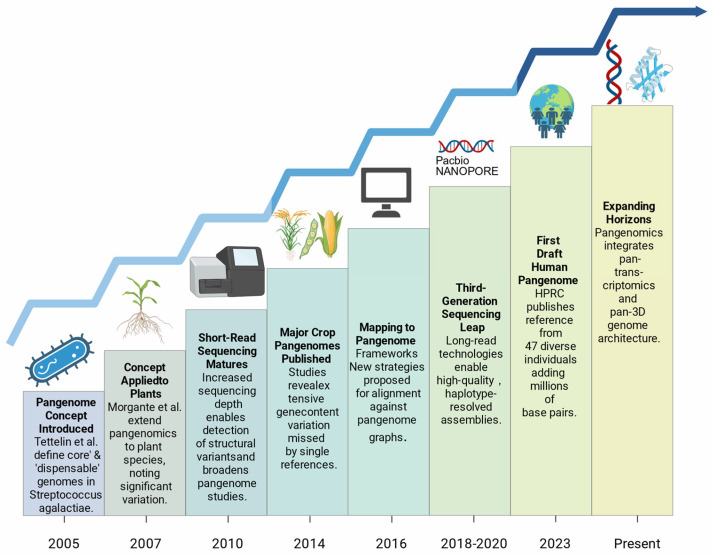
Key Milestones in the Development of Pangenomics. The figure outlines the major advances that have shaped pangenomics, including the emergence of the pangenome concept, the shift from single-reference genomes to population-scale genomic resources, the development of graph-based pangenomes, and the application of long-read sequencing, structural variation analysis, QTL/GWAS integration, and multi-omics approaches. Together, these milestones have expanded the role of pangenomics in genetic diversity analysis, trait interpretation, and agricultural breeding [5,9,10,11,12,13,14,15,16,17,18,24].

Pangenome research has now extended from prokaryotes to higher eukaryotes, transitioning from short-read linear models to long-read graph structures. With the maturation of T2T assembly technology, pangenomes have entered a new era of high-quality, comprehensive coverage, providing critical tools for deciphering genetic diversity and structural variation obscured by single reference genomes. As the technological foundation for revealing the genetic architecture of complex traits and supporting precision molecular breeding, the pangenome holds significant implications for biodiversity conservation and modern agricultural improvement. It is crucial to recognize that pangenome construction spans a methodological spectrum from reference-dependent to reference-agnostic approaches. While variant integration remains partially anchored to existing reference coordinates, its value is based on it being a pragmatic approach for exploring the resulting transformations in breeding technologies. The pangenomic path lies in its ability to integrate population-scale structural variants into a graph-based topology. This ensures a comprehensive representation of genetic diversity while maintaining computational efficiency for large-scale agricultural breeding. This paper systematically reviews pangenome construction methods and analyzes their role in trait analysis and the development of the pangenome, as illustrated in Figure 1.

The pangenome comprises a core genome and a variable genome. The core genome contains sequences shared across most individuals and often reflects conserved biological functions, whereas the variable genome captures lineage-specific sequences, structural rearrangements, and other dispensable components. Currently, three primary strategies are employed for pangenome construction: iterative assembly, de novo assembly, and graph-based pangenome.

Iterative assembly relies on high-throughput resequencing data, integrating unmapped sequences into a pangenome reference framework after alignment against a reference genome [25]. This approach is suitable for scenarios with existing high-quality reference genomes and large sample sizes. In 2018, researchers employed the “map-to-pan” method [26] to construct the rice pangenome. This approach identified 268 non-redundant novel sequences >500 bp in length, supplementing the Japanese rice reference genome. It predicted 12,465 new full-length genes and thousands of novel genes with partial sequences, providing support for subsequent rice genomics research and breeding [27].

Advancements in long-read DNA sequencing technologies, coupled with progress in bioinformatics, have revolutionized genomics. De novo assembly enables high-quality genome construction from deep sequencing of a small number of individuals, followed by comparative analysis to identify variable regions [28]. Accurate whole-genome alignment remains challenging for species characterized by large, highly repetitive, heterozygous, and polyploid genomes. Liu et al. [29] de novo assembled 26 soybean germplasm genomes from 2898 deeply sequenced individuals, revealing structural variations in thousands of candidate genes associated with agronomic traits. These variations, linked to domestication and evolutionary processes, offer novel perspectives for related research. Huang et al. [30] constructed a pangenome for fall armyworm and identified 1.37 Gb of non-reference sequences, highlighting significant intrapopulation genetic variation and offering new insights into its genomic evolution and the potential functions of genes acquired through horizontal gene transfer (HGT).

**Figure 2:**
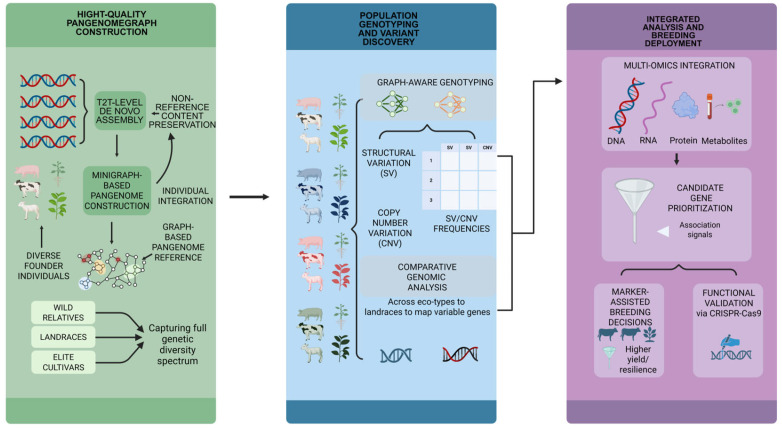
Pangenomics Technology Roadmap. Diverse germplasm or breed resources are sequenced and assembled to construct linear or graph-based pangenomes. These pangenomes capture core genes, dispensable genes, structural variants, presence/absence variations, copy number variations, and non-reference sequences that may be missed by single-reference genomes. Integration with QTL, GWAS, multi-omics, and phenotypic data facilitates trait interpretation, candidate gene discovery, marker development, and pangenome-assisted breeding.

Graph pangenomes utilize graph structures to represent sequence differences and variation pathways among individuals, intuitively reflecting the complex relationships between structural variation and PAVs [31]. Graph pangenomes extend the scope of variation investigation beyond the species level, opening new possibilities for studying the molecular and evolutionary mechanisms underlying speciation, selection, recombination processes, and adaptation to rapid climate change [32]. The pangenome workflow is illustrated in Figure 2.

## 2. Theoretical Foundations and Construction Methods of the Pangenome

The construction of graph pangenomes currently revolves around three primary technical approaches. The first is an integration strategy driven by known variants. This approach involves mapping population sequences back to a linear reference genome to extract variant call format (VCFs) files, which are then embedded into a graph topology using tools like vg [33]. Although this method exhibits exceptional computational efficiency when handling large-scale population SNPs and small-scale variations, it remains inherently constrained by linear coordinate systems, making it challenging to capture extensive non-reference sequences in highly diverged regions. Because VCF-based integration struggles with highly diverged regions, capturing extensive non-reference sequences requires shifting from read mapping to the direct alignment of de novo assemblies. For instance, whole-genome alignment (WGA) tools like Progressive Cactus [34] achieve precise cross-assembly sequence alignment by anchoring conserved coding regions, a strategy validated in pangenome studies of chili pepper [35] and rice [36], to resolve complex haplotype diversity. This necessity for robust assembly-to-assembly alignment naturally paves the way for the next two graph-construction paradigms, which rely on integrating complete genome assemblies rather than discrete VCF files. Although vg provides a genuine example of embedding known variants into a graph reference [33], this logic should not be extended to all tools used in the same workflow. AnchorWave improves whole-genome alignment in divergent or whole-genome-duplicated genomes [37], whereas SURVIVOR mainly merges, compares, simulates, and evaluates SV calls [38]. They can support variant discovery or preprocessing, but they are not themselves variant-integration tools.

Second is the reference genome-guided iterative construction paradigm. This approach aims to maintain coordinate consistency while dynamically expanding the pangenomic sequence landscape through de novo haplotype assembly. The Minigraph-Cactus workflow, currently the leading standard, combines the macro-stability of graph frameworks with single-base resolution alignment accuracy. By capturing SV skeletons via minigraphs [39] and supplementing with base-level alignment, this approach effectively addresses the limitations of linear reference genomes in characterizing complex genomic rearrangements [40]. In constructing the Human Pangenome Reference Consortium (HPRC), Liao et al. [24] employed this approach to reduce small-variant detection error rates by over 34%, demonstrating the iterative model-building method’s exceptional potential for enhancing variant calling reliability. The strength of reference-guided graphs is therefore conditional. The HPRC and Minigraph-Cactus studies show that graph construction can improve variant calling when haplotypes and benchmarks are carefully curated, but agricultural datasets often face more uneven assembly quality, stronger population structure, and less mature truth sets. This gap explains why a workflow validated in one context may not produce equivalent reliability in another.

The third major paradigm relies on a reference-unbiased graph construction strategy. To minimize reference bias, tools like pggb and Progressive Cactus employ decentralized or phylogenetically guided alignment logics, respectively. Specifically, by integrating wfmash, seqwish, and smoothxg [41], pggb adopts an all-to-all sequence alignment approach. While such reference-agnostic methods are not universally applicable across deeply divergent evolutionary lineages due to sequence decay, they authentically reconstruct the topological complexity of highly polymorphic regions among closely related species or diverse intra-specific accessions. Although exhibiting exponential growth in computational resource demands, this strategy demonstrates unparalleled completeness in elucidating complex structural variations underlying scientific propositions like grapevine domestication [42]. Reference-free all-to-all strategies reduce dependence on a chosen reference, but they do not automatically solve alignment ambiguity. For example, pggb and Progressive Cactus are valuable for assembly-to-assembly comparison, yet in large plant genomes with abundant repeats, polyploidy, or long evolutionary distances, graph complexity and fragmented alignment can become the limiting factors. This suggests that divergence, repeat architecture, and ploidy are key moderators of method performance.

Comparative assessment and practical method selection. No single strategy is universally optimal. Variant integration is attractive for large-scale genotyping projects that prioritize speed and compatibility with existing pipelines. Reference-guided iterative graphs are often a practical compromise when breeding programs require both coordinate stability and improved SV discovery. Reference-free de novo graphs are especially useful when the goal is to resolve deeply divergent haplotypes, cross-lineage structural evolution, or highly incomplete linear references. Table 1 summarizes the logic, representative tools, strengths, and major practical limitations of these mainstream strategies.

## 3. Pangenome-Enabled Trait Discovery and Biological Interpretation

The value of a pangenome is most evident when sequence diversity is translated into biological interpretation. Compared with single-reference analyses, pangenome resources make it easier to detect hidden variation, recover non-reference genes or regulatory elements, compare graph-supported haplotypes, and connect sequence architecture with trait variation across populations and species. To achieve this, variant integration should be recognized not as a mere extension of linear genomics, but as a foundational strategy that fulfills the pangenomic mandate of capturing hidden structural diversity. By transitioning from a single linear path to a multi-haplotype graph topology, this approach provides an inclusive pangenomic framework that encompasses both discovery-oriented de novo models and application-oriented integration models. In animal and plant species such as pigs, cattle, sheep, and chickens, pangenomes have elucidated the genetic basis of numerous traits related to meat quality, milk yield and key phenotypes [43], environmental adaptation [44], and body conformation traits [45]. Integrating pangenomes with QTL or GWAS data [46] effectively overcomes the limitations of single reference genomes by providing a more comprehensive genomic context. Rather than altering the statistical mapping resolution dictated by linkage disequilibrium within a population, this integration facilitates a more accurate biological interpretation of associated loci, allowing researchers to uncover the true causal variants—particularly complex structural variations—hidden within established GWAS peaks. The livestock and crop examples are connected by a common pattern but differ in interpretation. Pig and cattle pangenomes often emphasize breed-specific or introgressed structural variation, whereas crop pangenomes frequently highlight dispensable genes, resistance loci, or TE-mediated variation. This contrast suggests that the most informative variant class is not universal; it depends on domestication history, reproductive system, genome architecture, and the trait being measured.

### 3.1. Hidden Variant Discovery and Non-Reference Sequence Identification

The primary significance of pangenome construction lies in capturing extensive non-reference sequences overlooked by traditional linear models. In pig research, modern de novo assemblies and graph constructions of representative Eurasian populations [47] have initiated the discovery of previously neglected key mutations. Subsequent comprehensive pig pangenome analyses have robustly quantified this hidden diversity, successfully recovering 72.5 Mb of non-reference sequences [28,48]—representing a substantial genomic fraction absent from the standard reference assembly. Among these, 9 million base pairs are predominant in Chinese pig breeds, and a novel sequence within the fatty acid metabolism gene *TIG3* region was identified. Importantly, statements such as the 72.5 Mb recovered in the pig pangenome should be interpreted as species and dataset-specific estimates rather than universal constants. In avian research, Li et al. [49] employed high-depth assembly techniques to construct a chicken pangenome, identifying 1335 protein-coding genes and 3011 long non-coding RNAs not covered in the reference genome *GRCg6a*. These novel genes exhibited substitution rates three times higher than known genes, significantly updating the understanding of avian evolutionary rates. In the ruminant domain, the construction of bovine pangenomes [50,51] revealed 36–70 Mb of non-reference sequences, with approximately 18.9–30 Mb originating from yak genetic introgression. These sequences are enriched in key pathways, including immune response, olfactory receptors, and signal transduction. The broader conclusion is that the magnitude and functional distribution of missing sequence depend strongly on population diversity, sample size and the breadth of genetic diversity captured.

### 3.2. Genetic Architecture Analysis of Important Agronomic Traits

Pangenome-informed trait analysis has strengthened the identification of causal or near-causal variants for production, morphology, quality, and disease-related traits. Regarding production and quality traits, researchers identified significant haplotype differences in the pig *BTF3* gene between Eastern and Western pig breeds via graph pangenome analysis, establishing it as a key candidate gene regulating pork traits [52]. In poultry research, growth traits were confirmed to be closely associated with a deletion in the *IGF2BP1* promoter region on chromosome 27. This variation influences cell proliferation and metabolism by regulating the stability of target mRNAs [53,54]. Regarding reproduction and development, a pangenomic study of Meishan pigs identified the *PGR* gene encoding the progesterone receptor. This gene participates in responses to gonadotropin stimulation and plays a crucial role in enhancing reproductive capacity [55]. At the population level, an expanded analysis of Chinese cattle identified 156,000 non-redundant SVs and localized 206 SV enrichment hotspots covering approximately 195 Mb of functionally dense gene regions [19,56]. Mechanistically, these signals often operate by altering coding sequences, promoter architecture, chromatin accessibility, or local haplotype structure. For example, regulatory deletions or transposable element (TE) insertions may modify gene expression timing, whereas larger SVs can reshape dosage or disrupt long-range *cis*-regulation.

### 3.3. Environmental Adaptation Evaluation and Evolutionary Background Reshaping

Pangenomes provide a comprehensive perspective for tracing the evolutionary adaptations of domesticated animals in complex environments. In goat studies, pangenome analysis revealed a domestication-associated immune locus containing an extra copy of the galectin-9 gene cluster, reflecting a unique evolutionary feature of ruminants [57]. For high-altitude adaptation, researchers identified key genes, such as the porcine *KIT*, *EPAS1*, and *EGLN1* in pigs [52]. Analysis of environmental gradients across northern and southern Chinese cattle populations [58,59] precisely identified 2610 SVs exhibiting significant allele frequency divergence between distinct lineages. These variations are associated with 862 functional genes extensively involved in metabolic regulation and environmental adaptation. Furthermore, pangenome analysis revealed 1457 SVs in Chinese humped cattle populations likely originating from Javanese cattle, providing strong direct evidence for the polygenic origins and adaptive evolution of humped cattle [59]. Regarding evolutionary drivers, a pangenomic study of chickens indicates that the impact of hybridization on PAV content significantly outweighs genetic drift effects during domestication [60]. These results are especially informative for traits shaped by ecological gradients, such as heat tolerance, high-altitude adaptation, disease resistance, and resource-use efficiency. At the same time, interpretation must remain cautious because adaptive inference is sensitive to population sampling, graph completeness, and the distinction between selection signals and demographic history.

### 3.4. Integrative Research on Pangenome and QTL Mapping

Liu et al. [61] constructed a pangenome reference library for grapevine comprising 18 newly generated phased telomere-to-telomere assemblies and 11 previously published assemblies. From resequencing data of 466 grape varieties, they constructed a variation map containing 9,105,787 short variants and 236,449 SVs, mapping 148 QTLs for 29 agronomic traits (50.7% newly identified). Among these, 12 traits showed significant contributions from SVs. Li et al. [62] identified 456 Mb and 357 Mb of sequence based on the 1961 cotton pangenome. Using variant data, they identified 47 new SNP-based QTLs and 37 CNV-based QTLs, indicating genomic differentiation during cotton domestication and improvement. Tang et al. [63] mapped QTLs regulating seed oil content (SOC) across eight environments. By analyzing population transcriptomes of seeds, they identified 692 genes significantly associated with SOC and four gene modules. Within two QTLs, a pair of homologous genes, *BnPMT6*, was experimentally validated to negatively regulate SOC.

Dai et al. [64] found that European improved cattle possess significantly longer and more abundant runs of homozygosity (ROH) genomic segments indicative of recent shared ancestry, compared to Chinese and African cattle. These ROH regions are enriched with QTLs and genes associated with beef and milk production. These encompassed QTLs for carcass weight, average daily gain, body weight, and 12th rib fat thickness. Notably, these QTL regions harbor six key candidate genes, including *PLAG1*, *CHCHD7*, *MOS*, *LYN*, *TGS1*, and *TMEM68*. Furthermore, potential causal mutations and regulatory factors associated with genes within these ROH hotspots were identified. Expression quantitative trait locus (eQTL) signals were particularly abundant in multiple tissues, such as muscle, rumen, and testes in beef cattle, and fat and rumen in dairy cattle, corresponding to improvements in beef and dairy production. By integrating these multi-omic layers within a pangenome framework, researchers can leverage the expanded variation catalog to pinpoint functional variants that are absent from the standard reference. This integration is particularly helpful for distinguishing graph-supported causal candidates from the large background of neutral SVs and PAVs. It also improves the interpretation of eQTL-rich regions, promoter variation, and non-reference transcripts that would otherwise remain poorly anchored in linear-reference analyses.

### 3.5. From Association to Causality: Validation Burden and Reproducibility Challenges

Despite rapid progress, pangenome-based trait studies still face several recurring limitations. First, topological complexity in certain graph regions can create mapping ambiguities, leading to spurious variant calls (false positives). Simultaneously, the ranking of candidate genes often remains unstable across different bioinformatics pipelines, as results are highly sensitive to the specific choice of graph-construction algorithms and genotyping parameters. Second, results are often difficult to compare across studies because graph construction choices, sample composition, and genotyping algorithms differ. Third, the biological meaning of many detected SVs and PAVs remains uncertain without orthogonal validation, such as segregation analysis, transcriptomic support, targeted genotyping, genome editing, or functional assays.

**Figure 3:**
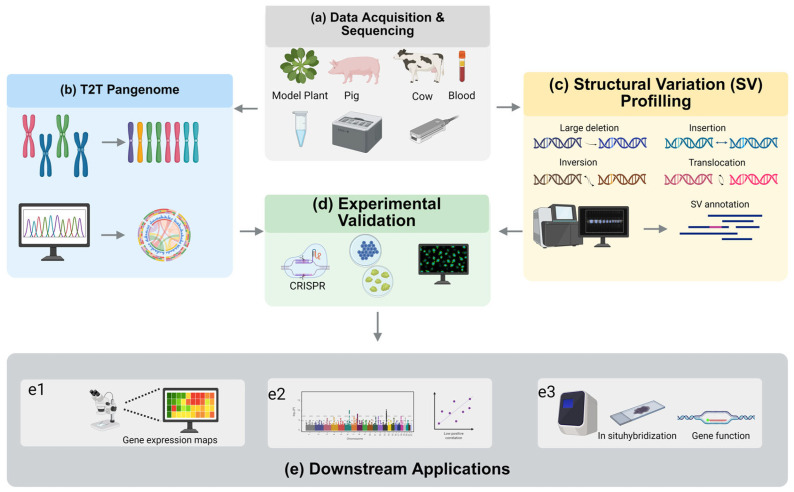
T2T Pangenomics Workflow for Structural Variation Characterization and Downstream Biological Applications. (**a**) Data acquisition and long-read sequencing; (**b**) T2T pangenome construction; (**c**) SV discovery and profiling; (**d**) Experimental validation using CRISPR-Cas; (**e1**–**e3**) Downstream biological applications and functional analysis.

These issues mean that pangenome studies should not be judged solely by the number of newly discovered variants. More informative benchmarks include the reproducibility of graph-aware genotyping, the proportion of candidates supported by independent evidence, and the extent to which identified variants can be converted into robust markers or experimentally testable hypotheses. Figure 3 outlines how high-quality T2T assemblies, graph construction, and downstream biological interpretation can be connected in a more rigorous analytical workflow.

## 4. Pangenome-Guided Breeding Framework

### 4.1. Pangenome Revealed Evidence of Adaptation and Evolution

Pangenome research profoundly illuminates evolutionary trajectories in complex habitats. In livestock research, a pangenome constructed from 21 pig components identified 105 Mb of non-reference sequences (NRS), revealing frequency differences in geographic adaptation between Eastern and Western pigs—particularly a significant insertion on the X chromosome in heat-tolerant breeds [48]. Similarly, bovine pangenome studies confirmed the impact of KIT gene translocation on coat color variation and identified core SVs associated with high-altitude adaptation, including *EPAS1*, *MB*, *DISC1*, *VWC1*, and *SNX3* [65,66,67], while confirming the importance of genes carrying variants like *ATPAF1* in fat tissue metabolism and evolution [46,68]. This mechanism is also present in plants. Plants exhibit similar mechanisms. The barley pangenome highlights divergent evolution in domesticated traits at loci governing disease resistance (*Mla*), plant architecture (*HvTB1*), and starch mobilization [69]. Concurrently, identifying 63 key structural variants in grapevine, such as *VvLHT8*, yields direct molecular targets to breed for improved water use efficiency, disease resistance, and stomatal traits [70]. Recent graph-based pangenomes also profoundly illuminate the evolutionary history of global staple crops. The *Aegilops tauschii* pangenome (the D genome donor of bread wheat) uncovers previously hidden disease resistance alleles, including the *Sr46* gene, by mapping a complex evolutionary history. This history stems from hybridizations among genetically and geographically discrete subpopulations [71]. Transitioning from species-level to genus-wide super-pangenomes further expands our grasp of crop evolution and environmental adaptation. The *Cicer* (chickpea) super-pangenome, for example, shows that structural variations within the dispensable genome strongly enrich for dispensable genes controlling crucial adaptive traits, including vernalization, flowering time, and disease resistance [72]. Similarly, a comprehensive potato pangenome spanning 296 accessions illustrates how transposable elements (TEs) within the cloud genome dynamically drive species evolution and adaptation to diverse environmental pressures and cultivation practices [73]. Peanut research revealed that *AhCKX6* and *AhARF2-2* regulate cytokinin content and protein interactions through insertions or specific fragment deletions in the 3′-UTR region, thereby driving seed enlargement [74].

Precise analysis of productive traits has enabled pangenomic mapping to overcome the limitations of traditional genomes in identifying complex variations. Regarding morphological development, *HOXB13* has been confirmed as the core locus regulating sheep tail length. By optimizing SV genotyping, researchers identified pathogenic mutations responsible for the long-tail trait, providing a basis for enhancing animal productivity [46,75,76,77]. Regarding economic yield traits, studies in pigs, geese, and ducks have identified key genes or transposable elements associated with fat deposition (*BRCA1*), body weight development (*TGFBR2*, *IGF2BP1*), and feather color formation (*MITF*) [78,79,80]. Furthermore, the convergent evolution of genes such as *BMPR1B* and *RALYL* in sheep and goats for wool fineness and meat quality traits further demonstrates the significant value of pangenomics in deciphering the genetic architecture of complex traits [81].

By integrating multidimensional data, including transcriptomes and methylomes, pangenome studies have further elucidated the fine-tuned regulation of phenotypes by epigenetic and non-coding elements. Research indicates that artificial selection directly modulates the expression timing of diachronous genes such as *GHSR* and *BDH1* during pig embryonic development by reshaping local DNA methylation patterns [81]. In avian species, the Gypsy TE was demonstrated to directly influence duck body size and plumage coloration by regulating promoter activity or affecting nascent transcripts [80]. Concurrently, non-reference networks constructed from pangenomes revealed thousands of novel genes and core/cloud gene distributions in species like domestic geese. These variations play crucial roles in key physiological processes, including reproductive capacity, feed efficiency, taste response (*TAS2R46*), spermatogenesis (*QRICH2*), and heat stress response (*HSPA1A*) [78,82,83].

Across species, current evidence already shows that pangenome-informed analyses can recover breeding-relevant variants that were previously hidden in linear-reference workflows. Table 2 and Table 3 summarize representative cases in animals and plants, including trait categories and the current level of validation. These examples support the translational value of pangenome resources, but they also show that most studies remain strongest at the discovery and prioritization stages rather than at routine breeding deployment.

### 4.2. Synergistic Application of Gene Editing and Pangenome Analysis

The use of CRISPR-Cas systems to regulate gene expression and conduct functional genomics has been extensively demonstrated [84,85]. Research has observed allele-specific phenotypes regulating disease resistance in rice [86] and oilseed rape [87]. Pangenome resources can also improve downstream validation and gene editing design. Because graph-based resources preserve alternative haplotypes and non-reference targets, they can reduce the risk that guide RNAs are designed against incomplete or non-representative reference sequences. This is particularly relevant when PAVs, promoter deletions, TE insertions, or allele-specific sequences alter the presence, copy number, or exact sequence context of editing targets. Concurrently, the accuracy of genome sequencing and annotation, along with the cost-effectiveness of related workflows, continues to improve. Huang et al. [88] integrated 66 rice genes associated with Green Revolution traits to construct a rice pangenome. SV analysis identified 29 conserved gene loci potentially linked to this shared phenotype. Subsequent CRISPR-Cas studies uncovered 31 genes correlated with high yield.

**Figure 4:**
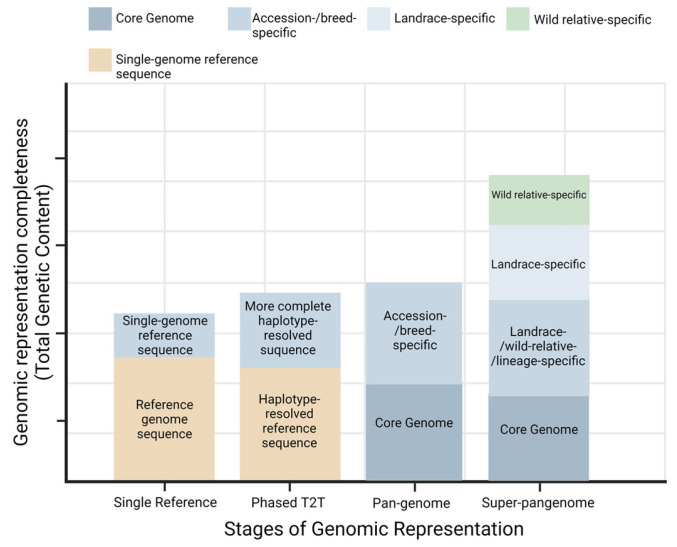
From Single Reference Genomes to Super Pangenomes in Animal and Plant Genomics. Single reference and phased T2T assemblies are shown as single-genome reference stages, whereas pangenome and super-pangenome resources incorporate broader population and lineage diversity. With expanded sampling, total genetic content increases, while the shared core genome decreases in relative proportion or stabilizes. Cultivar-, landrace-, and wild-relative-specific components represent additional diversity captured as sampling breadth increases. Bar heights and segment sizes are conceptual rather than quantitative.

Even so, the bridge from pangenome discovery to genome editing remains at an early stage in many agricultural systems, especially in animals. Most current studies still rely on pangenomes primarily for candidate discovery and marker development, whereas full graph-aware validation pipelines are much less mature. A realistic near-term path is therefore to use pangenomes to improve target prioritization and reduce design errors, while continuing to rely on conventional functional assays, population validation, and controlled editing experiments for causal confirmation (Figure 4).

## 5. Development Trends and Outlook

Future progress will depend less on simply increasing the number of assemblies and more on improving interoperability, benchmarking, and functional interpretation. Important priorities include open graph formats, stable graph versioning, standardized metadata, reproducible SV genotyping workflows, graph-aware annotation transfer, and benchmark datasets that include difficult regions and agriculturally relevant diversity. Marker conversion pipelines are a key translational bottleneck. Breeding programs need assays that are inexpensive, reproducible, and compatible with existing selection systems. Therefore, graph-derived variants must be filtered for genotyping reliability, population frequency, effect consistency, linkage with causal alleles, and assay design feasibility before they can be used in routine selection.

Biodiversity conservation is another important application. Pangenomes can reveal rare haplotypes, introgressed segments, locally adapted alleles, and lineage-specific sequences that are poorly represented in elite references. However, conservation conclusions require careful sampling and demographic interpretation; otherwise, rare sequences may be confused with sequencing errors, assembly artifacts, or under-sampled diversity.

Across both crops and livestock, the most useful future studies will be those that report not only new variants, but also evidence tiers: discovery only, replicated association, functional support, validated marker, or deployed breeding use. This evidence-based structure would make plant and animal pangenome studies more comparable and would reduce overgeneralization across species.

## 6. Conclusions

Pangenomes have substantially improved the representation of genetic diversity in agricultural species by recovering non-reference sequences, complex SVs, PAVs, and haplotypes that are often missed by single linear references. They have strengthened variant discovery, trait-region interpretation, adaptive inference, and multi-omics candidate prioritization in both animals and plants.

In conclusion, to maximize the utility of pangenome-guided breeding, it is essential to adopt an inclusive yet rigorous framework. Methodologically, variant integration should not be viewed as a mere extension of linear genomics, but rather as a foundational strategy that fulfills the pangenomic mandate of capturing structural diversity. By transitioning from a single linear path to a multi-haplotype graph topology, this approach ensures that pangenomics provides a practical analytical foundation. At the same time, pangenomics should not be overpresented as a direct route to precision molecular design. Its current strength lies in building more complete genomic representations and integrating evidence for candidate prioritization. Routine breeding deployment will require standardized construction workflows, transparent reporting, cross-platform benchmarks, validated markers, and experimental confirmation of causal mechanisms. A cautious, evidence-based framing will make pangenome research more useful for breeding than broad claims of improved precision without measurable benchmarks.

## Figures and Tables

**Table 1:** Comparison of Algorithmic Logic and Application Characteristics Among Three Mainstream Construction Strategies for Graph Pangenomes.

Strategy Development	Core Logic	Core Tools	Key Advantages	Limitations
Variant integration drive	Map the sample to the reference sequence and embed known variants into the graph via VCF files.	vg, AnchorWave, SURVIVOR	High computational efficiency; suitable for rapid analysis of SNPs and small-scale variations in large populations	Significant reference bias exists; it is difficult to capture large non-reference sequences or highly divergent regions
Iterative reference construction	Using the linear genome as a scaffold, non-reference sequences and structural variations are dynamically expanded through iterative alignment.	Minigraph-Cactus, minigraph	Balances coordinate consistency with structural variation capture; capable of generating base-level high-precision maps	Sensitive to the initial reference mass; The graphical structure is affected by the input sequence of the sample sequence
Build from scratch without reference	Fairly integrates all genomes through all-to-all alignment without requiring pre-selected reference sequences.	pggb, Progressive Cactus	Completely eliminate reference bias; accurately reconstruct evolutionary relationships among complex regions and distantly related species	Computational resource consumption is extremely high; The coordinate system is complex, making downstream functional analysis quite challenging

**Table 2:** Molecular Applications of Animal Pangenomes.

Species	Genes	Associated Traits	References
Pig	*TNFRSF19*, *BRCA1*, *GHSR*, *BDH1*	Heat resistance, fat deposition	[48,80]
Yak	*KIT*, *EPAS1*, *MB*, *DISC1*, *VWC1*, *SNX3*	Coat color, high-altitude adaptation, domesticability	[65,66,67]
Cattle	*ATPAF1*	Fat	[46,68]
Cattle	*TAS2R46*, *QRICH2*, *PRDM9*, *HSPA1A*	Taste response, spermatogenesis, gamete recombination, heat stress response	[82,83]
Sheep	*HOXB13*	Long Tail	[45,75,77]
Sheep	*BMPR1B*, *BMPR2*, *RALYL*, *COL21A1*, *LRP1B*	Capillarity	[81]
duck	*IGF2BP1*, *MITF*	Weight, coat color	[79]
Goose	*TGFBR2*	Weight	[78]

**Table 3:** Molecular Applications of Plant Pangenomes.

Species	Genes	Associated Traits	References
Barley	*Mla*, *HvTB1*, *amy1_1*, *HvSRH1*	Disease resistance, plant structure, starch mobilization	[69]
Peanuts	*AhCKX6*, *AhARF2-2*	Cell division, seed enlargement	[74]
Grapes	*VvLHT8*	Pathogen resistance	[70]
Chickpea	Dispensable genes (e.g., flowering loci)	Vernalization, flowering time, disease resistance	[72]
Potato	TEs	Species evolution, environmental adaptation	[73]

## Data Availability

No new data were created or analyzed in this study. Data sharing is not applicable to this article.
